# Successful Treatment of *Candida parapsilosis* Fungemia in Two Preterms with Voriconazole

**DOI:** 10.1155/2015/402137

**Published:** 2015-06-04

**Authors:** Emel Altuncu, Hulya Bilgen, Ahmet Soysal, Eren Ozek

**Affiliations:** ^1^Division of Neonatology, Department of Pediatrics, Faculty of Medicine, Marmara University, 34890 Istanbul, Turkey; ^2^Division of Pediatric Infectious Disease, Department of Pediatrics, Faculty of Medicine, Marmara University, 34890 Istanbul, Turkey

## Abstract

Herein, we report two preterms with invasive candidiasis refractory to liposomal amphotericin B (AMB) treatment in spite of low MIC levels (MIC: 0.5 mcg/mL). Both of the patients' blood cultures were persistently positive for *C. parapsilosis* despite high therapeutic doses (AMB: 7 mg/kg per day). After starting voriconazole blood cultures became negative and both of the patients were treated successfully without any side effects. In conclusion, although it is not a standard treatment in neonatal patients, our limited experience with these patients suggests that voriconazole appears to be a safe antifungal agent to be used in critically ill preterm infants with persistent fungemia despite AMB treatment.

## 1. Introduction

Critically ill newborns are candidates for systemic fungal infections. Despite the administration of amphotericin B (AMB), invasive candidiasis is sometimes complicated by persistent fungemia and refractory invasive disease [1]. The problem has been augmented by the increasing prevalence of nonalbicans species that are often resistant to fluconazole and AMB. Recent studies have expanded our knowledge of newer antifungal agents such as the second generation triazoles and the echinocandins in older children have given us opportunity to extend the range of therapeutic alternatives; however, little is known about their usage in neonates [2].

This report represents effectiveness of voriconazole in two neonates with invasive candidiasis refractory to AMB therapy.

## 2. Case Presentations

Case 1 was a 570 g male premature infant born at the 24th week of gestation by Caesarian section (C/S) from a mother with chorioamnionitis with Apgar scores 8, 9, and 9 at the 1st, 5th, and 10th minutes of life, respectively. The infant was intubated and placed on mechanical ventilation and received surfactant because of respiratory distress syndrome (RDS). An umbilical catheter was placed in the first hours of life. Systemic antibiotic therapy was initiated with ampicillin and gentamicin and they were stopped at the 7th day of life at the time of extubation.

On the 8th postnatal day, as the blood culture was positive for* Candida parapsilosis *the baby was placed on liposomal AMB (5.0 mg/kg/day) and the umbilical catheter was removed. Despite antifungal therapy, the patient developed persistent thrombocytopenia requiring platelet transfusions and liposomal AMB dosage was increased to 7 mg/kg/day. Cardiac, brain, and renal sonographic assessments and ocular examination of the infant were normal. At the 10th day of life, antifungal susceptibility test resulted in a low MIC (MIC: 0.5 mcg/mL) level for AMB. Despite higher AMB dosage and the removal of the central venous catheter, blood cultures remained positive for* C. parapsilosis* and at this point AMB was stopped and the systemic antifungal therapy was changed to voriconazole (8 mg/kg/day). The cultures obtained after 48 hours revealed negative blood/CSF/urine cultures for candida. Thrombocytopenia improved in a few days. The therapy was completed after 4 weeks without any side effects. Serial ultrasounds and fundoscopic examinations performed during and before treatment were normal. The infant was oxygen dependent at the corrected 36 weeks of age. His clinical course was also complicated by osteopenia of prematurity, anemia, apnea of prematurity, and clinical nosocomial sepsis. Retinopathy of prematurity (zone 2, grade 2) was detected during screening. The renal and liver function tests were within the normal limits during therapy. The patient was discharged on the 102nd day of life without any neurological and retinal sequelae. He is now 1 year old and his physical and neurological examination is normal.

Case 2 was a male premature infant born at 31 (+1) weeks of gestation via emergent C/S complicated by immune hydrops fetalis and fetal distress. His birth weight was 1750 g and the Apgar scores were 0, 1, and 4 at 1, 5, and 10 min, respectively. Urgent cardiopulmonary resuscitation was initiated and a silastic umbilical venous catheter was placed in the delivery room. The exchange/transfusion was performed with 0 Rh (−) packed red blood cells and whole blood in the delivery room.

The severely edematous baby was placed on mechanical ventilation and given two doses of surfactant six hours apart for RDS. The chest X-ray was remarkable for bilateral pleural effusions, pulmonary hypoplasia, and signs of fluid engorgement of the parenchyma. Pericardial effusion was detected during echocardiography which did not affect cardiac functions. Routine cultures of tracheal aspirates were negative at this time. Systemic ampicillin and gentamicin were started from birth. Albumin infusion and intravenous immunoglobulin were initiated immediately. Increasing doses of inotropes were required to maintain blood pressure in the normal range. The infant had renal and liver dysfunction and developed thrombocytopenia refractory platelet transfusions. The clinical course of the infant was complicated by grade 4 intracranial hemorrhage. During the first week of life, signs of acute renal failure (ARF) progressed and fluid overload was unresponsive to furosemide infusion. After the third exchange transfusion, ARF began to regress, urinary output gradually increased, and the infant began to lose weight. Throughout the first weeks, the infant required high settings on conventional mechanical ventilation.

Because of unimprovement in the respiratory status and persistent thrombocytopenia requiring frequent platelet transfusions, the antibiotic regimen was switched to cefepime, vancomycin, and fluconazole (FCZ) empirically on the 8th day. The other day it was reported that the blood culture yielded* C. parapsilosis *and FCZ was changed to liposomal AMB (7 mg/kg per day) (MIC 0.5 mcg/mL). Sonographic assessments including brain and abdominal sonographies and ocular examination for the potential seeding of the fungi were normal. Infected thrombus/vegetation in the mitral valve was detected by Doppler-echocardiography on the same day (postnatal 8th day). Because of the babies unstable condition, cardiac surgery was considered of high risk and treatment was continued with antifungal agents alone. As samples from blood cultures were repeatedly positive for* C. parapsilosis* and there were no reductions in the vegetations, AMBwas ceased and intravenous administration of voriconazole (8 mg/kg/day) was started.

After 48 hours of voriconazole therapy, blood cultures became negative, thrombocytopenia improved, and the progression of cardiac vegetations stopped. At the 40th day of life, he was extubated and enteral feeding was increased gradually. His clinical course was also complicated by osteopenia of prematurity, sepsis, and cholestasis, intracranial hemorrhage leading to hydrocephalus. Voriconazole was switched to its oral form after four weeks of therapy. The patient was discharged from the hospital on oral voriconazole therapy and therapy continued until the baby was one year of age. During the entire therapy, the liver and renal function tests were normal and no other side effects were detected. He is now 1 year old and has moderate neurological sequelae.

## 3. Discussion

Invasive candidiasis with non-albican species is the most frequent cause of death among neonatal infectious diseases and responds only to aggressive and prolonged antifungal therapy [1]. Amphotericin B or its liposomal formulations remain standard recommendations [3]. Due to treatment failures, the combination therapies with new antifungal agents, such as caspofungin and voriconazole, show promise in the treatment of candidiasis refractory to conventional therapy; however clinical experience with those new antifungals is limited in the newborn period [4]. Recently, Celik et al. reported that 12 newborns out of 17 with invasive fungal sepsis in whom the infection persisted despite conventional therapy had been cured successfully with voriconazole. In spite of cholestasis and liver function abnormalities drug was continued without permanent side effects [5].

Because of the persistent fungemia detected in our patients, antifungal therapy was changed to voriconazole. After the initiation of voriconazole, blood cultures became sterile within 48 hours of treatment.

Voriconazole, a triazole antifungal agent, acts via the inhibition of fungal ergosterol biosynthesis [4]. It is fungicidal against* Aspergillus* and active against all* Candida *species including* Candida krusei* and* Candida glabrata* [4]. It is available in both IV and oral formulations.

The first largest pediatric report of voriconazole was the evaluation of the drug in 58 children with proven or probable invasive fungal infection refractory to conventional therapies [6]. In their study, Walsh and colleagues described the efficacy (45% complete or partial response at the end of the therapy) and safety of the drug (less adverse reactions) in children [6]. Case reports also have documented the successful treatment of invasive neonatal candidiasis by voriconazole administered alone or in combination with other antifungals in a variety of conditions including those with meningitis [7], those with endocarditis [8], those with cutaneous aspergillosis [9, 10], and those with bloodstream infections [11, 12]. Turan et al. [13] reported 6 very low birthweight infants who had persistent candidemia despite antifungal treatment and who were treated successfully with voriconazole.

Generally, susceptibility studies indicate that most of the strains are susceptible to AMB as it was shown in our cases (MIC 0.5 mcg/mL). These cases reflect the difficulty in correlating data from in vitro susceptibility tests to microbiological improvement despite amphotericin B treatment. In both cases AMB had to be changed to voriconazole because of the persistence of positive blood cultures, although AMB dosage was increased from 5 to 7 mg/kg/day.

In conjunction with antifungal agents, aggressive surgical debridement, excision of localized infection, and removal of infected foreign bodies (such as intravenous catheters) are imperative to prevent or limit dissemination [14, 15]. Cardiac surgery in Case 2 was considered of high risk and we opted to treat him with antifungal agents alone. This therapeutic modality was reported successful in the neonatal age group with survival rates similar to those for combined medical and surgical treatment [15]. We have used IV therapy till the patient had to be in the hospital for other reasons and then completed the antifungal course with oral voriconazole therapy.

The adverse effects of voriconazole include fever, gastrointestinal symptoms, reversible visual disturbances, hepatitis, jaundice, and skin reactions [4]. In our cases, we did not observe any serious complications which might be attributable to voriconazole.

In conclusion, although it cannot be recommended as a standard treatment in neonatal patients based on the results of case reports with limited numbers, our experience with these patients suggests that voriconazole appears to be a safe antifungal agent to be used in critically ill preterm infants with persistent fungemia despite AMB treatment. Additionally, it has some advantages including wide spectrum of coverage without renal side effects and thrombocytopenia and a significant cost advantage over liposomal amphotericin. The oral preparation of the drug, for patients who need a longer duration of treatment, is another advantage.
